# HIV stigma in UK press reporting of a case of intentional HIV transmission

**DOI:** 10.1177/1363459320949901

**Published:** 2020-08-08

**Authors:** Rusi Jaspal, Brigitte Nerlich

**Affiliations:** Nottingham Trent University, UK; University of Nottingham, UK

**Keywords:** Daryll Rowe, HIV, HIV criminalisation, media, social representations theory, stigma

## Abstract

The UK has set itself the ambitious target of zero new HIV transmissions by 2030. HIV stigma is a significant barrier to achieving this target. Media reporting plays an important role in shaping social representations of HIV and of stigma. Between 2016 and 2018, the media in the UK reported on the Daryll Rowe case – the first criminal prosecution for intentional transmission of HIV in the UK. This article examines the way that UK newspapers reported this case, which may have exacerbated HIV stigma. Using Nexis, 178 UK newspaper articles were extracted and subjected to qualitative thematic analysis through a social constructionist lens. Informed by social representations theory, the analysis yielded three discursive themes: (1) Representing the perpetrator through HIV-focussed metaphors; (2) Constructing volitional ambiguity; and (3) Anchoring the lived experience of HIV to misery and death. UK newspapers constructed an ‘evil vs victimhood’ dichotomy in relation to Rowe and the men infected with HIV, respectively. This article argues that news coverage of the Rowe story constructs HIV in ways that are inconsistent with public health messaging. Reporting failed to note innovations in HIV treatment and prevention but instead disseminated stigmatising social representations of HIV. This is important because stigma impedes effective HIV prevention, engagement with HIV care and ultimately our ability to achieve the zero-infections target.

## Introduction

Between 2012 and 2018, HIV incidence in gay, bisexual and other men who have sex with men in the UK decreased by 71% (O’Halloran et al., 2019). The UK has now set itself the ambitious, but achievable, target to end all HIV transmissions by 2030. Combination prevention, consisting of condom use, treatment as prevention, pre-exposure prophylaxis and behavioural interventions, will be key to achieving this target (Hankins and Zalduondo, 2010). It is widely recognised that one of the most significant barriers to effective HIV prevention is the social stigma that surrounds the condition. HIV stigma inhibits accurate risk appraisal, regular testing, engagement with healthcare and, above all, open discussions about HIV (Valdiserri, 2002). Often, HIV stigma arises inadvertently from particular ways of representing and discussing the condition, especially in the media, but it can have insidious effects on prevention efforts. In this study, we examine social representations of HIV in media reporting of the first criminal prosecution of intentional HIV transmission, the Daryll Rowe case (Welsh, 2017). Drawing on social representations theory (Moscovici, 1988), we focus specifically on how HIV, its transmission and the experience of living with the condition are represented in the media and the potential implications of these representations for social behaviour and the ending of HIV by 2030.

### HIV and the media

Since 1981, 74.9 million people have been infected with HIV and 32 million have died of AIDS.^1^ The epidemic is relatively small in the UK, with an estimated 103,800 people living with the condition, which disproportionately affects gay, bisexual and other men who have sex with men (Public Health England, 2019). The association of HIV, the virus that causes AIDS, with gay sexuality, intravenous drug use and morality has resulted in significant stigma. On the one hand, this causes distress to those living with HIV and, on the other hand, stigma indirectly contributes to disease incidence (Valdiserri, 2002).

Yet, HIV medicine has developed significantly since 1981. In 1996, effective antiretroviral therapy was introduced which transformed HIV into a manageable chronic health condition. Moreover, antiretroviral therapy has progressively improved, many of the first-line drugs have few or no side effects, and some drug regimens consist of a single pill. In 2016, the use of tenofovir-emtricitabine was shown to be effective as pre-exposure prophylaxis to prevent HIV (McCormack et al., 2016). Furthermore, the INSIGHT START Study Group (2015) demonstrated that HIV patients who commence antiretroviral therapy soon after diagnosis (i.e. while their immune system is still relatively strong) exhibit much better long-term health outcomes than those who initiate antiretroviral therapy late. There is now evidence that the life expectancy of an individual diagnosed and treated early is near-normal. More recently, the second phase of the PARTNERS study (focusing on gay, bisexual and other men who have sex with men) demonstrated the efficacy of ‘treatment as prevention’ (Rodger et al., 2019).

The media have covered the HIV/AIDS pandemic extensively since the first clinical observations in 1981 (Jaspal and Nerlich, 2016, 2017; Labra, 2015; Lupton, 1994). Although much reporting has attempted to communicate the risks of HIV and the reality of living with the condition, often it is marred by scientific inaccuracy, social stigma and alarmism which in turn shapes public understanding and behaviour. Yet, the scientific developments in HIV medicine challenge the stigmatising social representations of fear, contagion and mortality which have long surrounded HIV. For instance, the discovery that a suppressed viral load removes the risk of HIV transmission (also referred to as ‘undetectable=untransmittable’ or ‘U=U’) has considerable potential to reduce HIV stigma as fear tends to be a key component of such stigma. However, it is doubtful that these scientific developments have received as extensive media coverage as rare, sensational and troubling criminal events, such as the Daryll Rowe case.

The media have the opportunity to increase public understanding of HIV, its transmission routes, and of the experience of living with the condition. Journalists have the ‘power to influence what society believes and should’ and, in principle, have a ‘responsibility towards society to provide truthful, balanced, factual and objective information’ (Champion, 2014; Hanne and Hawken, 2007). Yet, balancing power and responsibility can be challenging given that newspapers seek to sell copies and, thus, speed of reporting is of the essence. In such circumstances, it may be difficult to turn the focus of a story away from sensationalist personal accounts of people affected by an event (such as in the Daryll Rowe case) towards measured health communication, which is not, in itself, the task of the journalist. However, we want to highlight here that opportunities for such types of communication exist and that capitalising on them should be part of the responsibilities that journalists have towards society. In part, this article argues that taking such opportunities for factual information could be usefully incorporated into journalistic codes of practice because this has a crucial role to play in enabling us to achieve the zero-infections target by 2030. Alignment between public health messaging and journalistic reporting would be highly beneficial.

### The Daryll Rowe case

Daryll Rowe, originally of North Berwick, Scotland, was in his 20s when he was diagnosed with HIV in Edinburgh in April 2015. He refused antiretroviral therapy which would have reduced his HIV viral load to undetectable levels, thereby removing the risk of onward HIV transmission. Instead, Rowe reportedly used various pseudoscientific therapies, such as drinking his own urine, in an attempt to cure himself of HIV. Soon after his diagnosis, Rowe moved to Brighton, East Sussex, where, over a 5-month period, he engaged in condomless sex with at least eight different men whom he had met on gay social networking applications. In most cases, Rowe claimed to be HIV-negative and pressured his partners into engaging in condomless receptive anal intercourse. If they refused, he covertly removed the condom or tampered with the condom mostly by cutting off its tip. Rowe subsequently sent some of his sexual partners taunting text messages informing them that he was in fact HIV-positive and that he had removed or tampered with condoms – invariably after it was already too late for them to acquire post-exposure prophylaxis to prevent HIV.

In February 2016, Rowe was arrested and questioned by Sussex Police but later released on bail. He subsequently moved to Berwick-upon-Tweed, Northumberland, where he continued to engage in condomless sex with more men. He was finally arrested and charged with actual and attempted grievous bodily harm (GBH) in December 2016. On 15 November 2017, Daryll Rowe became the first person in England and Wales to be convicted of causing GBM under the Offences Against the Person Act 1861 for deliberately infecting, or attempting to infect, male sexual partners with HIV. He was sentenced to life imprisonment and will serve at least 12 years in prison before being considered for parole.

There have been a handful of criminal convictions for the sexual transmission of HIV in England and Wales and some of these cases, including that of Rowe, have been the subject of legal research (Weait, 2001, 2005; Welsh, 2017). Given its unprecedented nature, involving multiple individuals and with clear intention, the Daryll Rowe case was covered quite substantially in the media, unlike the other cases. Moreover, in view of the premeditated nature of his actions, there was a widespread desire to understand what had motivated him.

There has, so far, only been one article examining media reporting of this case as compared to another case in Italy (Carratalá, 2019). The author used quantitative and qualitative content analysis to study 81 pieces of news coverage (mostly extracted from UK newspapers). The study focused on comparing UK and Spanish media coverage of two cases of deliberate HIV transmission, rather than the broader social representations that the UK coverage generates. His findings showed that ‘coverage of the two trials privileges a sensationalist approach that upholds inaccuracies in the language used, and [. . .] is structured around the victim/villain dichotomy’ and he concluded that the mostly ‘emotional framing of the disease [. . .] can negatively affect how readers relate to HIV-positive people, in addition to revealing the persistence of certain homophobic biases in the media’ (p. 38). The study showed that the (gay) men whom Rowe infected were portrayed as victims and that HIV was represented as a disease affecting gay men, thereby attenuating HIV risk in other (at-risk) populations. Conversely, in our article, we explore the key social representations of HIV which were constructed, challenged and disseminated in UK newspaper reporting of the Daryll Rowe case, focusing in particular on the way this specific reporting sheds light on wider dilemmas affecting media reporting on HIV.

### Social representations theory

The media constitute a key source of societal information regarding science, medicine and health (Briggs and Hallin, 2016). In this study, we draw on social representations theory (Moscovici, 1988), which focuses on collective elaborations of knowledge and how cultural meaning systems evolve. A social representation consists of a network of ideas, values and practices in relation to a specific object – in this case, HIV. Social representations enable us to understand the novel and unknown through two principal social psychological mechanisms – anchoring and objectification:

- Anchoring refers to the process of making something unfamiliar understandable by linking it to something that we already know about. HIV may be linked to imagery of death and destruction, which in turn leads people to think of it as a life-limiting condition.
- Objectification is the process whereby unfamiliar and abstract objects are transformed into concrete and ‘objective’ common-sense realities – most notably through the use of metaphor – allowing us to map aspects of more familiar knowledge (the so-called source domain) onto more unfamiliar knowledge (the so-called target domain) (Lakoff and Johnson, 1980). For instance, the use of war metaphors (such as ‘weapon’ and ‘preying’) in relation to HIV transmission can serve to construct HIV as something that is volitionally transmitted in order to cause harm to others and may induce fear and, thus, stigma.

As an unprecedented legal case about a poorly understand health condition, the Daryll Rowe case is anchored to, and objectified in terms of, existing constructs in the popular media so that it resonates among readers and can become a topic for thought and discussion. Social representations theory enables the analyst to understand the origins, development and repercussions of emerging representations in this forum.

This study contributes to a vibrant tradition of research into social representations of infectious diseases from Sontag’s (1978, 1989) work on AIDS, its metaphors and social stigma to more recent investigations of infectious disease and society (Eicher and Bangerter, 2015; Herek, 1990; Jaspal and Nerlich, 2016, 2017; Washer, 2010). In this article, we focus on anchoring and objectification in the formation of social representations of HIV, its transmission and its lived experience in media reporting of the Daryll Rowe case. More specifically, we examine how these social representations may contribute to, or challenge, social stigma in relation to HIV, which constitutes a major barrier to its prevention.

The newspaper media may set the tone for public understanding about, and engagement with, such issues by influencing and reflecting policy agendas (Reese et al., 2003). They are therefore an important forum to study in order to understand emerging social representations of health issues. Our exploratory study of the Daryll Rowe case focuses on the UK print media, demonstrating in particular the discernible disconnect between stigmatising media reporting and public health messaging about risk, prevention and the reality of living with HIV in the era of effective antiretroviral therapy.

## Method

### The Corpus

Using the keyword ‘Daryll Rowe’ on the Nexis^®^ news database, we conducted a search of all national and regional/local newspapers in the UK and extracted all articles published between 26 February 2016 (the first article to be published on this case) and 30 September 2019 (the last article to be published). Using the ‘Hide Duplicate Results’ function on Nexis^®^, which excluded duplicate stories, this process yielded a total of 178 newspaper articles, all of which were relevant and therefore included in the analysis. We decided not to include articles published in the lesbian, gay, bisexual and transgender (LGBT) media, such as *Pink News* (in which 22 articles were published on the case), which tend to be aimed at, and consumed by, LGBT people. We were interested in the mainstream press and its role in shaping public understanding of HIV (in the general population).

The first articles to mention the case were published in *The Daily Record and Sunday Mail* (a Scottish tabloid newspaper based in Glasgow) and *The Herald* (a Scottish broadsheet newspaper). These outlets included in the analysis reflect a wide range of political perspectives (both right- and left-wing politics); feature both national and local newspapers; and are available both online and in hardcopy format. Table 1 provides an overview of the distribution of articles included in the analysis.

**Table 1.** Distribution of newspaper articles on the Daryll Rowe case.

Newspaper outlet	Number of articles
Daily Record and Sunday Mail	24
The Sun	23
The Herald	22
Scotsman	17
Daily Star	14
The Times	16
The Independent	13
Chroniclelive.co.uk	11
The Daily Telegraph	11
The Mirror	6
Evening Times (Glasgow)	5
The Western Mail	3
Aberdeen Press and Journal	2
Daily Post (North Wales)	2
The Express	2
The Belfast Telegraph	1
Liverpool Echo	1
Manchester Evening News	1
South Wales Echo	1
The Daily Mail	1
The Guardian	1
The Northern Echo	1
Total	178

### Analytical procedure

We analysed the corpus using a social constructionist variant of qualitative thematic analysis, which has been described as ‘a method for identifying, analysing and reporting patterns (themes) within data’ (Braun and Clarke, 2006: 78). This approach enables the analyst to present a fine-grained, micro-level analysis of linguistic representation, focusing on what is ‘being done’ with language, rather than the exclusive content of media reporting. This draws on Willig’s (2008: 112) observation that the discursive themes that arise from the analysis ‘facilitate and limit, enable and constraint what can be said, by whom, where and when’. Thematic analysis through a social constructionist lens enables the analyst to identify how the media construct particular social and psychological realities in its reporting and does not focus on any ‘objective’ reality or intentionality in reporting. Accordingly, this form of thematic analysis allowed us to identify emerging social representations of HIV, its transmission (by Daryll Rowe) and its lived experience (among those who acquired it). The dominant rhetorical strategies used to construct these social representations were a key focus of the analysis.

Both the headline and the main body of each article was subjected to thematic analysis. Images were not included in the analysis, as they are not available on Nexis^®^. We read and re-read the articles to familiarise ourselves with the broader themes that we subsequently discussed analytically. First, initial observations were made which captured the essential qualities of each article, the units of meaning, and dominant rhetorical techniques. Second, we discussed our respective initial codes, which included *inter alia* general tone, particular forms of language, comparisons, categorisations and emerging patterns in the data. Third, the initial codes were collated into preliminary themes, which reflected the content of the analysis, and subsequently arranged into a coherent structure that reflected the overall thematic analysis.

In addition to describing dominant themes in the corpus, we identified linguistic elements (especially metaphors), which performed the functions of anchoring and objectification. The superordinate themes can be considered social representations because they ‘assume a configuration where concepts and images can coexist without any attempt at uniformity, where uncertainty as well as misunderstandings are tolerated, so that discussion can go on and thoughts circulate’ (Moscovici, 1988: 233). In the analysis below, we provide extracts from the articles that exemplify the superordinate themes.

## Results

In this section, the following three discursive themes are described: (1) ‘Representing the perpetrator through HIV-focussed metaphors’; (2) ‘Constructing volitional ambiguity’; and (3) ‘Anchoring the lived experience of HIV to misery and death’.

### Representing the perpetrator through HIV-focussed metaphors

In the headlines of articles reporting the case, Rowe’s name was seldom used and was instead replaced by a variety of epithets which focused mainly on HIV. In some cases, he was simply referred to as ‘HIV man’^2^ and ‘HIV Scot’^3^ (referring to his Scottish national identity) with the elaboration that he had been ‘charged’,^4^ that he had ‘set out to infect more partners’, that he had ‘drunk his own urine’^5^ as a means of attempting to treat his infection, and that he had been ‘charged’ with a crime.^6^

In some cases, his occupation as a hairdresser was foregrounded invariably in conjunction with ‘HIV’, which was again used adjectivally: ‘HIV hairdresser’.^7^ The epithet ‘HIV hairdresser’ was frequently juxtaposed with judicial metaphors, such as ‘Key events that *brought HIV hairdresser to justice*’^8^ and ‘HIV hairdresser *freed on bail*’.^9^ Similarly, several headlines focused explicitly on his criminal status, as an ‘HIV suspect’,^10^ ‘HIV trial man^11^ ‘HIV fugitive’^12^ or as a ‘(serial) HIV attacker’.^13^ There was also some use of medicalised epithets, such as ‘HIV carrier’ which were juxtaposed with judicial metaphors such as ‘gets life’.^14^ Some articles referred to Rowe as an ‘HIV spreader’, and coupled this with the observation that he had been ‘jailed for life’,^15^ thereby establishing a connection between HIV transmission and life imprisonment (discussed in more detail below).

In most cases, however, ‘HIV’ was combined with animalistic metaphors, such as ‘predator’,^16^ ‘brute’ and ‘fiend’. The animalistic metaphor ‘fiend’ was used as the subject of the sentence ‘A fiend who infected lovers with HIV’^17^ and that of ‘brute’ was qualified with ‘guilty of infecting sex dates’.^18^ Moreover, in some articles, the animalistic metaphor that Rowe ‘preyed’^19^ on men was used to describe his actions. When used in relation to a human being, animalistic metaphors such as ‘fiend’ and ‘brute’ construct the individual as evil, devious and immoral. Indeed, there were several instances in which Rowe was described explicitly as a ‘devious and deceptive predator’,^20^ often through the discourse of key stakeholders in his case, such as the detective inspector and prosecutor involved in his case.

‘HIV’ was sometimes used in conjunction with ‘sex fiend’,^21^ thereby constructing a relationship between (gay) sex and Rowe’s behaviour. In the same article, this sexualising epithet was used alongside the notion that Rowe had a ‘porn secret’, that is, that he had featured in the gay adult porn films. There was an element of stigma appended to gay sexuality with one article referring to ‘unprotected romps’ between Rowe and the men whom he infected, and to Rowe as ‘lining up trysts’.^22^ This constructed the act of condomless sex as necessarily irresponsible and the use of geospatial social networking applications, such as Grindr, to seek sexual partners as clandestine. More generally, in attempting to explore the possible motives underpinning Rowe’s actions, one article noted that Rowe ‘came out as gay at the age of 15, in the same year that he experienced his first sexual encounter’,^23^ suggesting gay sexuality and early sexual debut as possible explanations for his behaviour.

Yet, HIV was not the only component of the descriptions used for Rowe – ‘virus brute’^24^ was also common in the corpus, as well as ‘Grindr monster’^25^ and ‘Date app fiend’.^26^ These epithets were often juxtaposed with other animalistic metaphors, such as ‘Virus brute *caged* in UK’,^27^ and with criminal metaphors, such as ‘HIV fiend *extra jail*’.^28^ In some cases, these labels were associated with the utterances of key figures in authority, such as Detective Inspector Andy Wolstenholme, who reportedly ‘blasted the monster’.^29^ Although the detective inspector did not actually use this word himself, its use with the detective inspector as the subject of the sentence served to distance the epithet from the journalist and to associate it with the detective inspector, thereby bestowing on the label a degree of legitimacy. In a further exploration of others’ responses to Rowe, the headline of an article focusing on other prisoners’ hostility towards Rowe after he was incarcerated reported ‘HIV fiend hounded from jail; lags target hated predator’.^30^ This article served to extend the animalistic metaphor of Rowe as a predator to that of other prisoners as now preying on him, thereby positioning him in a metaphorical field of the animal kingdom. Furthermore, the representation that convicted criminals ‘hate’, ‘hound’ and ‘target’ Rowe further legitimised use of stigmatising terms to describe him.

‘HIV’ was also used as part of a number of verbal compounds to describe Rowe’s actions. Judicial metaphors were used alongside ‘HIV’ and included ‘HIV rampage’,^31^ ‘HIV GHB charges’,^32^ ‘HIV sex charges’,^33^ ‘HIV offences’^34^ (for which Rowe was reportedly ‘charged’), ‘HIV violence’^35^ (for which he was reportedly ‘jailed’) and ‘HIV attacks’.^36^ These might be referred to as ‘compound crimes’ which in turn serve to anchor HIV transmission to criminal activity (focusing on physical violence) and to judicial consequences (i.e. criminal charges). Similarly, articles which focused on the judicial case that followed Rowe’s actions used HIV-focused compounds, such as ‘HIV court case’^37^ and ‘HIV life sentence’,^38^ thereby reiterating the anchoring of HIV to judicial consequences and entrenching negative connotations of the illness. Throughout the corpus, there was, unsurprisingly, a focus on Rowe’s crime but, in most newspaper headlines, the crime was poorly defined and the real focus shifted to HIV, equally ill-defined.

### Constructing volitional ambiguity

Most article headlines in the corpus were characterised by ‘volitional ambiguity’ in relation to the crime, that is, ambiguity about the degree to which Rowe intentionally attempted to infect his sexual partners with HIV. Rather than stating *volition* explicitly, there was a tendency for ambiguity and many of the headlines, in particular, left open the possibility that HIV transmission was non-volitional, that is, unintended.

Article headlines were often very ambiguous, referring simply to a ‘man held over HIV infection’^39^ or a ‘man charged over HIV’,^40^ without elaborating on the nature of his crime or focusing on the notion of intentional HIV transmission. Here, unlike the HIV compounds discussed above, the focus was squarely on the illness or infection, framing it as a criminal tool, even weapon. In another article, the epithet ‘HIV infection accused’^41^ which constructed his crime as the transmission of HIV to others, without clarifying the circumstances under which HIV transmission had occurred or, crucially, the degree to which the act of transmission had been volitional. In several articles, it was noted that Rowe (typically depersonalised in terms of a ‘hairdresser’ or ‘man’) was ‘accused of *giving* lovers HIV’^42^ and that he ‘gets second jail term for *giving* partners HIV’.^43^ The verb ‘to give’ in relation to HIV transmission was neutral and could include non-volitional HIV transmission, but, in this context, it rather came to mean ‘inflicting’. Similarly, in other articles, it was observed, sometimes through the discourse of the prosecutor, that Rowe ‘was HIV positive’ and that his offences involved ‘him sleeping with his alleged victims’.^44^ This served to construct the act of having sex with others (here, referred to as ‘victims’) when living with HIV as a criminal offence, rather than the intentional transmission of HIV to unsuspecting sexual partners.

In some articles, a contrast was drawn between Rowe ‘who infected victims with HIV’ and others’ perception of him as ‘a genuine person’.^45^ In the absence of more information about his case, this contrast between action (‘infecting victims’) and self-presentation (as genuine) suggested that HIV transmission is necessarily indicative of disingenuity towards ‘victims’, that is, unsuspecting individuals. The headline foregrounded the social representation that HIV transmission is volitional and the constructed intentionality in HIV transmission extended beyond the specific case of Rowe. Thus, HIV transmission was anchored to disingenuity, thereby suggesting a sinister motive underpinning the transmission of HIV to others.

In most article headlines, the act of HIV transmission was anchored to judicial metaphors. For instance, the specific observation was often made that Rowe had been ‘*charged* over HIV infection’,^46^ that he ‘*pleads guilty* to infecting victims’,^47^ and that he had been ‘*jailed for life* for infecting men with HIV’.^48^ Moreover, there was reference to a ‘*manhunt* for a *suspect*’ which culminated in ‘cops making an arrest’.^49^ The anchoring of HIV transmission to judicial metaphors served to construct HIV transmission invariably as a criminal act and to establish a discursive connection between the act of transmitting HIV and judicial retribution.

The article headlines referred to the indirect object, that is, the individuals who acquired HIV as a result of Rowe’s actions, in a variety of ways. In some articles, they were referred to as ‘victims’, which suggested, but did not confirm, volitional HIV transmission. The reference to these individuals anchored HIV acquisition to victimhood, thereby suggesting that the perpetrator is invariably the person who is living with HIV and transmits it. Moreover, as discussed in the next subsection, the emphasis on victimhood constructed the experience of living with HIV in adverse terms. In other articles, the anchoring of HIV transmission to judicial metaphors appeared with ‘men’,^50^ ‘people’,^51^ and ‘Grindr dates’^52^ as the indirect object. Moreover, there was sometimes no reference to those who acquired HIV as a result of Rowe’s actions – one headline reported, ‘man jailed for spreading HIV’.^53^

### Anchoring the lived experience of HIV to misery and death

Although the majority of media reporting focused on Rowe, there was consistent reference to the health and wellbeing of the men who acquired HIV as a result of Rowe’s actions. The men’s reported experiences (mainly through direct quotes from the men themselves) were often represented as evidence of that Rowe was an evil person. A consistent tendency across the corpus was the emphasis of the men’s victimhood and, accordingly, they were referred to as ‘HIV victims’,^54^ which can be contrasted with the HIV-focussed epithets used to describe Rowe. Several articles used the judicial metaphor that the men were ‘living with a life sentence’,^55^ which referred to their HIV infection. Furthermore, in describing the men’s experiences in the aftermath of their encounters with Rowe, the media focused on both the physical and psychological experience of living with HIV, both of which were represented negatively as being conducive to significant psychological distress. Articles referred to ‘having to live with the “devastating consequences” of contracting the virus’,^56^ which constructed HIV invariably as being distressing.

Several articles referred to HIV as ‘the deadly virus’^57^ or a ‘deadly sexual disease’^58^ which anchored HIV to imagery of death. This is consistent with dominant social representations of HIV in the pre-treatment era but inconsistent with modern developments in medical science which ensure a good prognosis and normal life expectancy if one is diagnosed and treated early. Furthermore, a quote from one of the men who acquired HIV was reproduced in order to illustrate the lived experience of HIV: ‘I felt like I had been left with a poison inside me’,^59^ which referred to his infection with HIV. Alongside anchoring to death imagery, use of the poison metaphor objectified HIV in terms of a life-limiting condition.

Similarly, another man who acquired HIV contrasted his lived experience of HIV with that of victims of other forms of GBH: ‘While for some cases of GBH it might be possible for victims to put the acts behind them, unfortunately this will never be the case for me. . . There is a virus inside me which will give me a horrible and painful death unless I take pills for the rest of my life’.^60^ His experience of GBH (that is, his acquisition of HIV) was constructed as more serious, enduring and indeed life-limiting than other forms of GBH which reportedly might be more easily tolerated. Conversely, his acquisition of HIV was anchored to death imagery, which was qualified as ‘horrible and painful’. In other words, HIV was positioned as being even worse than death due to the suffering that it could cause before death. Similarly, another individual was quoted as saying ‘it [HIV] was a lifelong sentence, which would eventually kill me off’, further anchoring HIV to death imagery.

Most articles focusing on the lived experience of HIV also emphasised the psychological trauma experienced by the men who had acquired HIV and constructed their overall experience of living with the condition as psychologically distressing. The psychological distress was attributed not to the specific circumstances of their acquisition of HIV (namely, through intentional transmission by Rowe) but rather to the experience of living with the condition *per se*. A quote from one of men (reproduced in several articles) constructed HIV infection as a rupture in his sense of continuity, that is, between past, present and future: ‘the old me is no longer. The new me is constantly sad, thinking about how my life has changed’.^61^ The construction of discontinuity is consistent with the aforementioned anchoring to death imagery since the ‘old me’ was said to be defunct and to be replaced by an undesirable ‘me’.

An article included a quote from another individual, which constructed HIV as destructive to his life because of the stigma associated with his condition: ‘I just felt like my life had been ruined, and that nobody would want to know me. There is still a huge stigma to HIV and I thought that no men would want to come near me’.^62^ This quote foregrounded the social representation that individuals living with HIV are treated as social pariahs, which is questionable in the advent of decreasing HIV stigma and ‘U=U’. Furthermore, this quote served to construct the experience of living with HIV as socially isolating and, thus, the destruction metaphor ‘ruined my life’ was used to describe his experience with the condition. Similarly, another individual noted that ‘Rowe claimed he was clean so we didn’t use condoms. Then I caught HIV. He has ruined my life’.^63^ In this quote, first, the individual used the term ‘clean’ to refer to being HIV-negative which implied that those living with HIV are, conversely, dirty. This clearly accentuated the stigma surrounding HIV. Second, Rowe was said to have transmitted HIV to him and, thus, ‘ruined’ his life, which anchored HIV to destruction beyond repair.

In view of both the physical and psychological trauma to which HIV was anchored, articles also represented a proclivity for the men who acquired HIV to contemplate suicide. A victim was quoted as saying that his diagnosis felt like being ‘hit by a bus’ and that ‘you feel like your life is over. You feel like you should go to a high place and jump’.^64^ Another was quoted as referring to HIV as having a ‘shattering effect’^65^ on his life. The experience of being diagnosed with HIV was constructed as being worse than death itself – indeed, an individual was quoted as saying ‘I would rather he had murdered me than left me to live my life like this’.^66^ This suggested that the experience of living with HIV rendered life unbearable, thereby representing death as preferable to living with HIV. Similarly, some of the men were said to be contemplating suicide: ‘many told how they had considered suicide, having suffered physical and psychological damage, needing to take daily medication’.^67^ One of the men was quoted as saying, ‘I think about committing suicide most of the time. Daryll has destroyed my life. I lost a lot of weight and felt very ill for a long time. I can’t see myself having a relationship ever again’, while another reportedly said, ‘I think about committing suicide most of the time. Daryll has destroyed my life.’^68^ The consistent anchoring of HIV to death and destruction imagery in the corpus contributed to the social representation that HIV is worse than death.

Most articles in the corpus focused on representing Rowe as malevolent through the use of negative epithets. This tendency appeared to be the discursive priority which superseded the discussion of innovations in medical science that have transformed HIV from a life-limiting into a life-changing illness. All but one of the articles in the corpus failed to mention innovations in medical science – the one article that did directly challenged the legitimacy of outlining these innovations in the context of Rowe’s case: ‘. . .the arguments advanced by his [Rowe’s] defence team give cause for concern. Felicity Gerry QC claimed that HIV was “not a terminal illness”, stating: “Those that live with HIV have good and high life expectancies. There is a need for therapy and not incarceration.”’^69^ The article proceeded to invite readers to ‘Imagine the horror these poor men lived through in court, only to have a smart QC claim their lives were going to carry on as before, belittling their situation’.

Thus, innovations in HIV medicine were constructed not only as irrelevant in this context but also as insult to the men who contracted HIV as a result of Rowe’s actions. The focus was clearly on representing Rowe as evil, his compound crimes as malevolent and the men who acquired HIV as victims. The social representation that Rowe is evil overshadowed broader public health messages about HIV prevention and the reality of living with HIV in the era of antiretroviral therapy. Within this discursive context, the social representation that HIV is worse than death was able to thrive.

## Discussion

In our study, we show that there is an overarching focus on communicating the evil of Daryll Rowe, the perpetrator of the crime, on the one hand, and on emphasising the victimhood and plight of those who acquired HIV as a result of his actions, on the other hand. There were three main social representations which were constructed and drawn upon in order to substantiate this ‘evil vs victimhood’ dichotomy in media reporting of the case: first, there was a social representation that HIV is a social, moral and legal flaw; second, HIV was socially represented as a criminal tool; and, third, the corpus constructed a social representation that HIV is a psychologically, emotionally and physically destructive disease. These three discursive themes demonstrated the social and linguistic mechanisms (principally anchoring and objectification) which were used in order to construct and legitimise the three social representations. These included representing Daryll Rowe using negative epithets with HIV-focussed metaphors; ambiguity in the level of volition underlying his actions; and anchoring the lived experience of HIV to misery and death.

### Accentuating HIV stigma

There has been a focus on reducing HIV stigma in the UK with some success. The National AIDS Trust (2014) found small gradual improvements in attitudes towards people living with HIV in three surveys conducted in 2007, 2010 and 2014. However, these small gradual improvements in attitudes could be undermined by stigmatising reporting of high-profile cases, such as that of Daryll Rowe, which attract media attention and, thus, reintroduce social representations of a relatively poorly understood health condition, such as HIV. They may provide the interpretive ‘resources’ to increase and substantiate HIV stigma in the general population.

Indeed, the use of HIV-related metaphors and compounds to construct negative epithets, which replaced Daryll Rowe’s name in article headlines, appended animalistic characteristics to Rowe, dehumanised him and constructed his brutality (Vaes et al., 2012). Yet, HIV was foregrounded in these epithets and a connection was established between the virus and the negative traits that the articles purported to associate with Rowe. More specifically, animalistic, judicial and war metaphors were used in conjunction with HIV in order to construct negative epithets in the article headlines. These included the construction of Rowe as an ‘attacker’, ‘fugitive’ and him being ‘caged’ as a result of his actions. In some cases, the stigma of gay sexuality was accentuated and drawn upon in order to frame the perpetrator negatively, whose identity was dominated by HIV imagery (Carratalá, 2019), which means that representations of a dangerous man became conflated with a disease that is not necessarily dangerous any more. In short, it is HIV that was being emphatically negativised alongside Rowe as the perpetrator – indeed, Rowe was seldom named in headlines as his name was replaced by these negative HIV-focussed epithets.

Furthermore, the article headlines were generally concise but rather sensationalist and used negative phrases constructed with HIV-focussed metaphors and compounds without specifying the level of volition underlying Rowe’s action. This served to obscure whether it was the act of transmitting HIV or doing so intentionally that was deplorable. Indeed, such ‘volitional ambiguity’ was a recurrent theme throughout much of the media’s reporting of the case, which may engender uncertainty about HIV criminalisation laws. HIV criminalisation is a controversial topic with some arguing that this is necessary to protect people and others arguing that its effects are counterproductive, serving only to stigmatise HIV further and to lead people to avoid it rather than discuss it openly (Mykhalovskiy, 2015). The discursive tendency to obscure volition served to construct *HIV* (rather than deliberate transmission) as a criminal tool, which was further bolstered by the animalistic, judicial and war metaphors that were frequently used in conjunction with HIV in reporting. Yet, it is noteworthy that most infections occur because people are actually unaware of their HIV status and, thus, transmit HIV when they are untreated and have a high viral load (Jaspal and Bayley, 2020).

Many articles focussed directly on the ‘victims’ of Rowes’ ‘HIV’ crime, that is, using and spreading a ‘deadly virus’. Readers were exposed to dramatic personal stories about the negative impact of being infected with HIV and subsequently living with the condition. The feelings expressed by the victims hark back to the beginning of the AIDS crisis, when treatment was not yet on the horizon and when living healthily with HIV was, as yet, unheard of. Those infected by Rowe referred to suicidal thoughts, to death and misery, and to lives being ruined, shattered and wrecked irreparably. The metaphor of random destruction was prominent. HIV was anchored to irreparable damage to health, rather than to a treatable illness. Some even constructed HIV as a poison residing inside their bodies slowly destroying them. This reflects the tension between public health messaging which seeks to prevent HIV but also to promote psychological wellbeing in HIV patients who can now live and thrive with the condition.

Unlike Carratalá’s (2019) study, we show how the accounts of the men who acquired HIV from Rowe (and the metaphors of death and destruction used in them) contribute to the social representation that HIV is a psychologically, emotionally and physically destructive disease. We show that HIV itself (not the man who deliberately transmitted the virus) was the focus of stigma. Moreover, a key finding from our study was that there was just one invocation of the scientific developments in HIV medicine which now render HIV a manageable chronic condition but that these developments were challenged and constructed as irrelevant and insulting to the victims. Thus, in contrast to Carratalá’s (2019) study, ours shows that the social representation of HIV as invariably destructive prevails.

### The disconnect between media reporting and public health messaging

It is important for the media to be specific in reporting, to recount the event accurately and to communicate the voices of victims. However, it is arguably also important to seize the opportunity to communicate broader public health messages surrounding HIV, its transmission and the experience of living with the condition in view of medical developments (see also Jaspal and Nerlich, 2017). Given their status as a principal source of societal information concerning health, science and medicine for many people (Briggs and Hallin, 2016), the media have the potential to perform a significant public health function.

Yet, none of the newspaper articles challenged the social representation that HIV is a psychologically, emotionally and destructive disease by providing the right information in the right way, which could bring hope into the lives of those living with HIV and motivation to those who have yet to test for it. This representation of HIV as invariably and inevitably fatal may increase feelings of victimhood and stigma and lead to thoughts not only of suicide but also of social isolation. Indeed, poor mental health (including suicidal ideation) remains a key comorbidity of HIV infection (Jaspal and Bayley, 2020).

In his study, Carratalá (2019) notes that reporting of the Rowe case may attenuate risk in the general population. Our findings suggest, more specifically, that the fatalist representation of HIV may engender fear in relation to HIV testing and promote denial and disengagement, rather than engagement which will be key to ending HIV transmissions by 2030. Moreover, the anchoring of personal experiences to ‘suicide’ and ‘destruction’ potentially poses challenges to identity – particularly self-esteem, sense of continuity and self-efficacy (Breakwell, 1986). HIV was represented as shattering identity, psychological wellbeing and life itself – it reportedly leads to a loss of self, to self-stigmatisation, and to death and destruction. This may undermine public health messaging concerning the possibility of living well with HIV if one engages with HIV care.

An analysis of media reporting of HIV (National AIDS Trust, 2015) found that almost half of all media coverage in 2014 focussing on people living with HIV was negative compared to that of people living with testicular and cervical cancer and that, in the news reports, there was often a focus on how the individual had contracted the disease and on blaming them (as reckless or risky) for their infection. Even in UK media reporting on potentially positive developments in HIV prevention, such as post-exposure prophylaxis and pre-exposure prophylaxis (Jaspal and Nerlich, 2016, 2017), there was polarised reporting which accentuated HIV stigma. On the one hand, people at risk of HIV were represented as being reckless and, on the other hand, the prevention approaches as facilitating reckless behaviour. It is noteworthy that there tend to be good or bad news stories about HIV in the media and previous research shows that even the good news stories (e.g. on effective HIV prevention) do not necessarily dispel HIV stigma. Our study focuses on an important case – that of Daryll Rowe – which attracted significant media attention and facilitated the reproduction of stigmatising representations also observable in previous media studies.

Having said this, one has to acknowledge that journalists find themselves grappling with a dilemma, namely writing sensationalised and headline-grabbing stories that, intentionally or not, amplify stigma, or counterbalancing such reporting with messages that attenuate this trend which runs counter to public health messages. This is not easy, but we suggest that journalists should at least be made aware of the dilemma that they face in HIV reporting.

### Limitations

There are some limitations, which should be addressed in future research. First, our study focused on media reporting in the mainstream national and regional press, though it is acknowledged that the case was also covered in the LGBT press, such as in *Pink News*. It would be advantageous to examine how the case was reported on these media platforms, which tend to take a more critical view of HIV stigma, because HIV stigma also remains a significant challenge in the gay community (Jaspal and Bayley, 2020). Second, our article examined social representations in one possible forum in which social representations are constructed, disseminated and challenged – the print media. Future research should also examine how these representations are also taken up on other platforms, such as on social media, where they may further influence public understanding. Third, the Daryll Rowe case generated significant media attention. Further research should explore media reporting of other developments in HIV science, such as the emergence of antiretroviral therapy and treatment as prevention, to corroborate our finding that the media tend to focus on and, thus, fuel HIV stigma. Fourth, although our study identified a disconnect between media reporting and public health messaging on HIV prevention, it is not possible to discern empirically the impact of reporting on public understanding. Future research ought to use experimental methods to show the impact of media reporting of this case.

## Conclusion

This article argues that news coverage of the Rowe story constructed HIV in ways that are inconsistent with public health messaging. Reporting reinforced dominant social representations of HIV in the pre-treatment era but did not acknowledge, or explain, modern developments in HIV science which ensure a good prognosis and normal life expectancy if one is diagnosed and treated early. None of the articles noted the advent of antiretroviral therapy, pre-exposure prophylaxis or ‘U=U’, all of which play a key role in reducing HIV stigma and in promoting effective HIV prevention and wellbeing. Media reporting of the Rowe case understandably focused on the events, the motivations of Rowe, and the voices of those he infected. However, social stigma is a recurring motif which may have significant implications for how people think, talk and behave in relation to HIV and, consequently, for our ambition to prevent new HIV transmissions by 2030.

## Notes

1.
https://www.who.int/gho/hiv/en/

2. The Herald, 3/11/2017

3. The Herald, 25/10/2017

4. Evening Times, 26/12/2016

5. The Herald, 3/11/2017

6. The Times, 26/12/2016

7. Daily Star, 4/05/2018

8. The Scotsman, 4/11/2018

9. The Daily Telegraph, 18/11/2017

10. Daily Mirror, 26/12/2016

11. Daily Record, 7/11/2017

12. Daily Record, 25/11/2016

13. The Herald, 21/11/2018

14. The Daily Telegraph, 19/04/2018

15. Daily Mirror, 19/04/2018

16. The Sun, 5/05/2018

17. The Sun, 1/09/2018

18. The Sun, 16/11/2017

19. The Herald, 18/11/2017

20. The Herald, 5/05/2018

21. The Sun, 9/05/2018

22. The Sun, 5/05/2018

23. The Herald, 19/04/2018

24. The Sun, 19/04/2018

25. Daily Record, 5/05/2018

26. The Sun, 16/11/2017

27. The Sun, 19/04/2018

28. The Sun, 5/05/2018

29. The Sun, 16/11/2017

30. The Sun, 1/09/2018

31. The Sun, 19/04/2018

32. Daily Mirror, 27/12/2016

33. The Sun, 27/12/2016

34. The Daily Telegraph, 26/12/2016

35. The Western Mail, 19/04/2018

36. Daily Record, 7/02/2017

37. Evening Times, 7/02/2017

38. The Daily Record, 2/11/2018

39. The Herald, 26/02/2016

40. The Times, 26/12/2016

41. The Herald, 13/10/2017

42. The Herald, 27/12/2016

43. Daily Telegraph, 5/05/2018

44. Daily Mirror, 27/12/2016

45. The Scotsman, 21/11/2018

46. The Western Mail, 26/12/2016

47. The Scotsman, 4/05/2018

48. Daily Mirror, 19/04/2018

49. Daily Star, 25/12/2016

50. The Independent, 18/04/2018

51. Daily Star, 25/12/2016

52. The Scotsman, 15/11/2017

53. Belfast Telegraph, 2/11/2018

54. The Times, 17/05/2018

55. The Independent, 18/04/2018

56. The Express, 19/04/2018

57. The Sun, 1/09/2018

58. The Independent, 18/04/2018

59. The Scotsman, 21/11/2018

60. The Independent, 18/04/2018

61. The Scotsman, 18/04/2018

62. The Scotsman, 21/11/2018

63. The Sun, 9/05/2018

64. The Independent, 16/11/2017

65. The Independent, 18/04/2018

66. The Sun, 19/04/2018

67. The Independent, 18/04/2018

68. The Times, 19/04/2018

69. The Independent, 20/04/2018
